# Effectiveness of tailored talks between a cancer screening specialist and general practitioners to improve the uptake of colorectal cancer screening in Ancona (Italy) during the pandemic period

**DOI:** 10.1080/13814788.2024.2340672

**Published:** 2024-04-15

**Authors:** Cecilia Acuti Martellucci, Giusi Giacomini, Maria Elena Flacco, Lamberto Manzoli, Margherita Morettini, Mosè Martellucci, Sara Rosati, Silvia Bizzarri, Matthew Palmer, Lidia Pascucci, Marco Uncini, Francesca Pasqualini

**Affiliations:** a Department of Environmental and Prevention Sciences, University of Ferrara, Ferrara, Italy; b Oncologic Screening Unit, Ancona Healthcare Agency, Ancona, Italy; c Department of Medicine and Surgery, University of Bologna, Bologna, Italy; d Department of Biomedical Sciences and Public Health, University of the Marche Region, Ancona, Italy; e The Daffodil Centre, University of Sydney, A Joint Venture with Cancer Council NSW, Sydney, Australia; f Melbourne School of Population and Global Health, The University of Melbourne, Melbourne, Australia

**Keywords:** Colorectal cancer, cancer screening, general practice, Italy

## Abstract

**Background:**

Colorectal cancer (CRC) screening uptake in many countries has been low and further impacted by the COVID-19 pandemic. General Practitioners (GPs) are key facilitators, however research on their impact on organised CRC screening is still limited.

**Objectives:**

To evaluate the effectiveness of tailored talks with GPs to increase population uptake of the long-established CRC screening programme in Ancona province, Italy.

**Methods:**

In this prospective cohort study, one-to-one tailored talks were organised in January 2020 between the GPs of one county of the province (with GPs from other counties as controls) and the screening programme physician-in-chief to discuss the deployment and effectiveness of organised screening. Data was extracted from the National Healthcare System datasets and linear regression was used to assess the potential predictors of CRC screening uptake.

**Results:**

The mean CRC screening uptake remained stable from 39.9% in 2018–19 to 40.8% in 2020–21 in the 22 GPs of the intervention county, whereas it statistically significantly decreased from 38.7% to 34.7% in the 232 control GPs. In multivariate analyses, belonging to the intervention county was associated with an improved uptake compared to the control counties (+5.1%; 95% Confidence Intervals – CI: 2.0%; 8.1%).

**Conclusion:**

Persons cared for by GPs who received a tailored talk with a cancer screening specialist avoided a drop in CRC screening adherence, which characterised all other Italian screening programmes during the COVID-19 emergency. If future randomised trials confirm the impact of tailored talks, they may be incorporated into existing strategies to improve population CRC screening uptake.

## KEY MESSAGES

Tailored talks on CRC screening were conducted between one cancer screening specialist and GPs.

Even during the pandemic, CRC screening uptake was stable among persons cared for by GPs targeted by tailored talks.

If confirmed by randomised trials, tailored talks may be employed to improve CRC screening uptake.

## Introduction

In 2020, colorectal cancer (CRC) was the second most common malignancy in women and the third most common in men and caused an estimated 915,880 deaths worldwide [1]. Organised CRC screening has reduced mortality in many countries, including Italy [2], where most provinces offer biannual faecal immunochemical testing (FIT) [3]. However, screening uptake has often been below the 45% acceptability threshold the Italian Group for Colorectal Cancer Screening set even before the COVID-19 emergency [4]. Uptake has been further worsened by the impact of the pandemic, which caused significant delays in screening activities [5].

Almost all European countries started CRC screening in the year 2000; most used FIT and a few adopted colonoscopy [6]. The overall national uptake in Italy has been stable over the last decade, at about 33% while only a few European countries reached 45%, according to the latest standardised statistics [5,7]. Indeed, in 2022 CRC screening uptake was 35% and 41%, respectively, in France and Spain and reached 66% and 68%, respectively, in the United Kingdom and the Netherlands [8–11]. Normally, general practitioners (GPs) are not involved in first-level CRC screening procedures, although some programmes automatically add the GPs’ signature to invitation letters [12]. Additionally, most European programmes notify GPs of a positive FIT result, so that GPs are able to monitor these patients, prompting them to undergo the second-level test where needed [13]. Aside from this, further collaborations between the GPs and screening programmes were only reported in studies which assessed interventions to increase uptake, such as sending GPs reports about the screening rates among their eligible persons or reminders to direct patients to perform screening tests [14,15]. In the majority of such assessments, involving GPs resulted in an increased uptake [13].

GPs may play an important role in improving patients’ adherence to screening programmes in their practices [16]. However, evidence is still scarce on strategies that motivate GPs to promote screening. To date, only two French studies, one cohort study and one cluster randomised controlled trial [17,18], showed a significant increase in the screening uptake after training sessions for GPs. The present prospective historical cohort study evaluates the potential impact on CRC screening uptake of a pilot intervention designed to strengthen the promotion of the screening programme by GPs.

## Methods

### Setting

In the province of Ancona, Italy, the CRC screening programme recommends and provides free of charge, biannual FIT to all citizens aged 50-69 years, residing or domiciled in the province, and registered with a GP practice. Invitations are sent by letter and FIT vials can be retrieved at any pharmacy, brought back to 34 collection points, and analysed by one laboratory. The GP’s signature is automatically added to the invitation letter in this pathway. However, GPs are otherwise involved only upon the request of the individuals: eligible persons may contact their GP to enquire after being screened or may be reminded during a routine GP visit. Historically, the province has not been able to reach the minimum 45% uptake threshold, and therefore, the Direction of the Local Health Unit has requested the implementation of corrective measures [19]. Consequently, the Oncologic Screening Unit performed a pilot study evaluating the feasibility of an intervention for increasing the motivation of GPs to promote CRC screening. This intervention aimed to increase CRC screening uptake among GPs’ registered persons who are eligible for screening.

### Design and recruitment

In this prospective cohort study, we included all GPs from the province of Ancona in service from January 1 2018 to December 31 2021 and with more than 20 eligible persons. We defined as ‘eligible persons’ all the individuals registered within the practice of a single GP who were eligible for CRC screening but who did not necessarily interact with the GP as part of the intervention. GPs working in Fabriano county were included in the intervention group, while the GPs working in the rest of the province constituted the control group. This choice was not based on any sample size calculation but rather on the personnel and time resources available to the province’s screening programme, as Fabriano county is the smallest in the province.

### Intervention

The intervention was structured as follows: in January 2020, the chief physician of the screening programme, a specialist in Oncology, conducted single, one-to-one tailored talks (lasting about 30 min) with all GPs of Fabriano county. These were only 22, mostly organised in practices of two or more GPs. Therefore at least four talks per day were conducted, which allowed 100% coverage of the county GPs within one month. During these meetings, the screening expert described the effectiveness, aims and procedures of the CRC screening programme. Subsequently, the expert prompted the GPs to discuss the potential critical steps of the screening pathway and the strategies for improving screening uptake. After the meeting, no reminders were sent, and further discussions were initiated if the GPs requested more information.

The main topics explored during these meetings were: (1) the effectiveness of CRC screening programmes and the importance of increasing adherence; (2) the eligibility of persons for FIT testing within the screening programme; (3) the guarantee to schedule a follow-up colonoscopy within 30 days of a positive FIT, which is unavailable to people tested outside of the programme; (4) the potential improvements in communication between the GPs and the screening programme.

### Data collection

After anonymisation, data was retrospectively extracted from the cancer screening dataset of the Local Health Unit of Ancona and referred to the biennium 2018-19 (pre-intervention) and 2020–21 (post-intervention). It should be noted that although FIT execution was suspended from March 11 to July 31, 2020, due to the COVID-19 emergency, all those eligible for CRC screening were invited during the 2020–21 biennium. For each GP, we retrieved the age, gender, geographical area of the office, and, for both periods, the number of persons who were eligible for CRC screening and participated in it, and the total number of persons who were simply eligible for screening participation.

### Data analysis

We computed the CRC screening uptake (defined as the percentage of persons undergoing screening out of the eligible ones) separately for each cluster of persons registered with single GPs, and also the difference in uptake from 2018–19 to 2020–21, comparing the Fabriano county with the rest of the Ancona province [20]. To evaluate the potential association between the proposed intervention and the variation in screening uptake, we used a random-effects linear regression model using geographic area as the clustering variable. We adjusted for all recorded variables: GPs’ age, gender, the variation in the number of eligible persons across the two periods and the mean number of eligible persons. Statistical significance was defined as a two-sided *p* value <.05 for all analyses performed using Stata 15.1 (Stata Corp., College Station, TX, USA, 2017).

### Ethics

The tailored talks were carried out as part of the routine activity of the Local Health Care Unit, while the retrospective data collection and the data analysis were approved by the Ethics Committee of the Marche Region on April 1, 2020, with number 2020–84. Informed consent was waived due to the secondary nature of the data and of the large number of investigated persons.

## Results

### Study sample

The final sample consisted of 254 GPs, who assisted 100,467 eligible persons in 2020–21. In 2018, the mean age of GPs was 58.2 ± 7.5 years, and male GPs represented 63% of the sample. Twenty-two GPs belonged to the Fabriano county (intervention group, with *n* = 7780 eligible persons in 2018–19) and 232 to the rest of the province (control group, with *n* = 84,546 eligible persons).

### Outcomes

In the intervention arm, the mean uptake increased from 39.9% (*n* = 3080) in 2018–19 to 40.8% (*n* = 3642) in 2020–21 while it decreased from 38.7% (*n* = 31,196) to 34.7% (*n* = 31,544) in the control group (Table 1). The proportion of GPs who reached the 45% acceptable threshold of uptake doubled (from 9.1% in 2018–19 to 18.2% in 2020–21) in the intervention county, whereas it decreased by 32.0% (from 22.8% to 15.5%) in the control group. From 2018–19 to 2020–21, the mean number of eligible persons grew in both groups, increasing from 354 to 404 in the intervention county (+12.7%) and 364 to 395 (+8.4%) in the control area.

**Table 1.** CRC screening uptake across GP clusters in fabriano vs the rest of the ancona province. Periods 2018–19 and 2020–21.

GPs-characteristics	Overall (*n* = 254 GPs)	Intervention county (*n* = 22 GPs)	Control counties (*n* = 232 GPs)	p* (between groups)
Male gender, %	63.0	63.6	63.0	.9
Age in 2018 in years, mean (SD)	58.2 (7.5)	61.2 (5.9)	58.0 (7.5)	.012
*Years 2018*–*19*				
Number of eligible persons, mean (SD)	363 (137)	354 (80)	364 (142)	.6
% uptake, mean (SD)	38.8 (10.8)	39.9 (8.4)	38.7 (11.0)	.5
GPs reaching the 45% uptake threshold, %	21.6	9.1	22.8	.13
*Years 2020*–*21*				
Number of eligible persons, mean (SD)	396 (88)	404 (58)	395 (90.1)	.9
% uptake, mean (SD)	35.2 (9.8)	40.8 (6.4)	34.7 (9.9)	.003
GPs reaching the 45% uptake threshold, %	15.7	18.2	15.5	.7
% difference in eligible persons 2020–21 to 2018–19, mean (SD)	8.8 (26.1)	12.7 (13.7)	8.4 (27.0)	.2
*p*^†^ (within groups)	<.001	<.001	<.001	
Difference in % uptake 2020–21 to 2018–19, mean (SD)	−3.6 (8.4)	0.9 (8.6)	−4.0 (8.3)	<.001
*p*^†^ (within groups)	<.001	.6	<.001	
Difference in % GPs reaching the 45% uptake 2020–21 to 2018–19, %	5.9	9.1	−7.3	.03
*p*^†^ (within groups)	.007	.3	.001	

CRC: colorectal cancer; GP: General Practitioner; SD: Standard deviation.

*Kruskal–Wallis test for continuous variables and Chi-squared test for categorical ones for comparisons between groups.

^†^Wilcoxon matched-pairs signed-rank test for continuous variables and Exact McNemar’s test for categorical ones for comparisons within groups.

The county-by-county analysis revealed that the difference between the intervention county and the rest of the province was mainly driven by the low uptake of Ancona county, which is also the largest: 34.1% in 2018–19 to 28.5% in 2020–21 (Table S1). Nevertheless, the intervention county remained the only one where uptake did not decrease in 2020–21.

### Multivariate analysis

After adjusting for GPs’ age, gender, and number of eligible persons, the variation of the CRC screening uptake was significantly higher in the intervention group, as compared to controls (+5.1%; 95% Confidence Intervals – CI: 2.0%; 8.1%; Table 2). A sharper decline in eligible persons in 2020-21 compared to 2018–19 was also independently associated with a higher increase in screening uptake +1.7% (95% CI: 1.2%; 2.1%) for each 10% decrease in the number of patients. Finally, no significant differences in uptake were observed by GPs’ gender while a 10-year increase in age was associated with a+ 1.5% (95% CI: 0.2%; 2.9%) variation in screening uptake.

**Table 2.** Results of the random-effects linear regression predicting differences (%) in CRC screening uptake in 2020–21 compared to 2018–19, with geographic area as the cluster variable.

GPs-characteristics	Coefficient	95% CI	p*
Fabriano (vs the rest of the Ancona Province)	5.07	2.02; 8.11	.001
Male gender	0.89	−1.00; 2.78	.4
Age (10-year increase)	1.51	0.16; 2.85	.028
Difference in eligible persons 2020–21 to 2018–19, (10% decrease)	1.65	1.22; 2.07	<.001
Mean number of eligible persons from 2018–21, (100-persons decrease)	0.24	−0.76; 1.24	.6

CRC: colorectal cancer; CI: confidence interval. *Two-tailed Wald test.

Each talk lasted approximately 30 min, plus an average of 20 min for travel time. When considering also the 30 min spent by the GPs for the tailored talk, the total time required by the intervention amounted to one hour and 20 min for each GP. No extra time was considered as the laboratory processing of samples is automatic and the opening times of FIT vials collection points was unchanged.

## Discussion

### Main findings

This study offers evidence of the potential impact of interventions aiming to improve the motivation of GPs to promote CRC screening uptake. It is the first Italian study to assess the possible effect of a single-contact communication intervention [21]. The main finding was the positive association between the participation of GPs in single-contact, one-to-one tailored talks with a screening expert and the change in the uptake of CRC screening among their eligible persons. Notably, GPs who took part in tailored talks witnessed a slight increase in screening uptake among their eligible persons despite disruptions in healthcare caused by the COVID-19 pandemic and the significant rise in the number of eligible persons of each GP [22], which both resulted in a generalised decrease in screening uptake observed in the province and in other regions of Italy for other cancer screening programmes [23,24].

### GP characteristics and impact on population uptake of CRC screening

Although interactions between GPs and their eligible persons were not recorded in the present assessment, our findings are likely explained by the influence that GPs are known to exert on their patients’ healthcare choices and are in agreement with only two published studies [25], both French, that evaluated the potential effectiveness of talks directed to GPs, focusing on CRC screening programmes [17,18]. Both the non-randomised and the randomised study found increases in CRC uptake among the people assisted by GPs who underwent training on communication about CRC screening [17,18], however, the settings of the two studies had a baseline uptake below 30%, by far lower than most European screening programmes, suggesting that there likely was a wider margin for improvement [7]. A further study from Australia recently tested an SMS intervention delivered through GP practices, showing a 16.5% increase in uptake compared to the persons that did not receive the SMS [26].

In the present work, the slow replacement of retired physicians determined a sharp increase in the mean number of persons assisted by each GP, which showed an inverse association with uptake. This is consistent with the single Italian study available to date, from the Lazio region, while in disagreement with the only other similar evaluation [21], from the USA [27]. However, the setting of the latter study was characterised by incentives for the quality of care, while no financial incentives were granted to the GPs in the present Italian study or in the previous [21,27]. Indeed, research suggests that having more registered persons likely decreases GPs’ attention to each individual [28]. Similarly, it should be noted that more prominent cities, like Ancona county in this study, were consistently found to have lower screening uptake in Italy and England [29,30]. On the contrary, rural areas tended to show lower screening uptake in the USA and Australia, where remoteness is presumably a more significant barrier to accessing healthcare [31,32].

A notable finding was the independent association between GPs’ age and the variation in CRC screening uptake. Indeed, the persons assisted by older GPs showed a higher increase in participation. This was observed for the first time and may be partly explained by the lower baseline uptake showed by the persons assisted by older GPs (35.9%) as compared to those assisted by the younger GPs (41.3%).

Finally, the exemption of the intervention county from the generalised fall in screening uptake observed throughout Italy and internationally during the COVID-19 emergency is an additional indication of the impact of the tailored talks [5]. Indeed, in 2020 compared to the previous year, invitations and tests carried out in Italy decreased by respectively 20% and 25% while in other organised CRC screening programmes worldwide, this drop in invitations ranged from 1.3% to 40.5% [5], leading to an estimated 7900 preventable deaths between 2020 and 2050, in the absence of catch-up [33,34]. As mentioned, the catch-up of the invitations backlog was performed within the year 2021 in the study setting. However potential missed diagnoses and preventable deaths will have to be assessed in the context of larger, multicentre studies, over the next years.

### Strengths and limitations

The study’s strengths include the use of official certified data on the entire resident population, with a very low (< 5%) proportion of missing, privately performed FIT. Although monocentric, this study included a large sample of over 100,000 persons eligible for CRC screening.

The study has some limitations that must be considered. First, the study design is observational, and the findings require confirmation through randomised studies. Second, we could not collect data on individual-level determinants of screening such as socioeconomic status. However, within the Ancona province it is unlikely that the disparities across GP clusters were significant enough to explain the observed difference between the intervention and control counties, as the average income does not substantially vary across counties [35]. Third, the small number of GP-level variables prevents assessing potential screening uptake determinants beyond gender, age, and cluster size. Similarly, it was not possible to assess any characteristics of the eligible persons since the software of the screening programme did not allow for extensive collection of such data of all persons included in the analysis (over 100,000). Finally, no systematic evaluation was conducted to investigate the methods used by GPs to counsel their eligible persons about CRC screening, which should also be the object of further studies.

## Conclusion

This study indicated that a single intervention, namely one-to-one tailored talks between a cancer screening specialist and GPs, may significantly improve population CRC screening uptake. Naturally, GPs’ influence is limited to the persons who consult them and since GP-patient interactions were not monitored, further research is required to confirm the effectiveness of the proposed intervention. A cluster-randomised trial is currently being planned with GP practices as a cluster unit. If the results are confirmed, personalised interventions involving GPs might be integrated among the other strategies used or recommended to improve CRC screening uptake.

## Supplementary Material

Supplemental Material

## Data Availability

All data is available from the corresponding author upon reasonable request.
