# Supplementary material for: Effectiveness of tailored talks between a cancer screening specialist and general practitioners to improve the uptake of colorectal cancer screening in Ancona (Italy) during the pandemic period
Source: Eur J Gen Pract. 2024 Apr 15;30(1):2340672. doi: 10.1080/13814788.2024.2340672 (PMC11020593; doi:10.1080/13814788.2024.2340672)
Supplement: Supplemental Material [file IGEN_A_2340672_SM9098.doc]

**Table S1.** CRC screening uptake across GP clusters in Fabriano vs the other counties of the Ancona province. Periods 2018-19 and 2020-21.

| **GPs-characteristics** | **Fabriano county**  **(*n* = 22)** | **Senigallia county**  **(*n* = 46)** | **Jesi**  **county**  **(*n* = 56 s)** | **Ancona**  **county**  **(*n* = 130)** | ***P** (between groups)** |
| --- | --- | --- | --- | --- | --- |
|  |  |  |  |  |  |
| Male gender, % | 63.6 | 69.6 | 66.1 | 59.2 | 0.6 |
|  |  |  |  |  |  |
| Age in 2018 in years, mean (SD) | 61.2 (5.9) | 58.7 (6.5) | 57.2 (8.3) | 58.0 (7.5) | 0.08 |
|  |  |  |  |  |  |
| *Years 2018-19* |  |  |  |  |  |
| Number of eligible persons, mean (SD) | 354 (80) | 374 (149) | 340 (123) | 371 (146) | 0.28 |
| % uptake, mean (SD) | 39.9 (8.4) | 43.8 (9.3) | 45.3 (9.3) | 34.1 (10.8) | <0.001 |
| GPs reaching the 45% uptake threshold, % | 9.1 | 39.1 | 42.9 | 8.5 | <0.001 |
|  |  |  |  |  |  |
| *Years 2020-21* |  |  |  |  |  |
| Number of eligible persons, mean (SD) | 404 (58) | 391 (87) | 374 (91) | 405 (90) | 0.14 |
| % uptake, mean (SD) | 40.8 (6.4) | 41.5 (7.7) | 43.6 (5.5) | 28.5 (7.2) | <0.001 |
| GPs reaching the 45% uptake threshold, % | 18.2 | 23.9 | 42.9 | 0.8 | <0.001 |
|  |  |  |  |  |  |
| % difference in eligible persons 2020-21 - 2018-19, mean (SD) | 12.7 (13.7) | 6.1 (25.6) | 9.2 (23.9) | 8.9 (28.8) | 0.24 |
| *P*^†^ (within groups) | <0.001 | 0.28 | 0.007 | 0.001 |  |
|  |  |  |  |  |  |
| Difference in % uptake 2020-21 - 2018-19, mean (SD) | 0.9 (8.6) | - 2.3 (7.9) | - 1.6 (7.2) | - 5.6 (8.6) | <0.001 |
| *P*^†^ (within groups) | 0.6 | 0.055 | 0.10 | <0.001 |  |
|  |  |  |  |  |  |
| Difference in % GPs reaching the 45% uptake 2020-21 - 2018-19, % | 9.1 | -15.2 | 0.0 | -7.7 | 0.027 |
| *P*^†^ (within groups) | 0.3 | 0.020 | 0.9 | 0.002 |  |
|  |  |  |  |  |  |

CRC = colorectal cancer. GP = General Practitioner. SD = Standard deviation. *Kruskal-Wallis test for continuous variables and Chi-squared test for categorical ones for comparisons between the Fabriano county and each control county ^†^Wilcoxon matched-pairs signed-rank test for continuous variables and Exact McNemar's test for categorical ones for comparisons within groups.
